# Exosomal IDH1 increases the resistance of colorectal cancer cells to 5-Fluorouracil

**DOI:** 10.7150/jca.58846

**Published:** 2021-06-11

**Authors:** Hao Yang, Sha Xie, Beibei Liang, Qiqi Tang, Huanchen Liu, Dongliang Wang, Gang Huang

**Affiliations:** 1 Shanghai Key Laboratory of Molecular Imaging, Shanghai University of Medicine and Health Sciences, Shanghai 201318, China.; 2 Department of Nuclear Medicine, Ren Ji Hospital, Shanghai Jiao Tong University School of Medicine, Shanghai, 200127, China.

**Keywords:** colorectal cancer, 5-Fluorouracil, IDH1, exosomes

## Abstract

Chemoresistance challenges the clinical treatment of colorectal cancer and requires an urgent solution. Isocitrate dehydrogenase 1 (IDH1) is a key enzyme involved in glucose metabolism that mediates the malignant transformation of tumors. However, the mechanisms by which IDH1 is involved in colorectal cancer cell proliferation and drug resistance induction remain unclear. In this study, we found that IDH1 was highly expressed in human colorectal cancer tissues and could be used to indicate a high-grade tumor. *In vitro* gene overexpression and knockdown were used to determine whether IDH1 promoted the proliferation of the colorectal cancer cell line HCT8 and resistance to 5-Fluorouracil (5FU). Further studies have shown that the 5FU-resistant cell line, HCT8FU, secreted exosomes that contained a high level of IDH1 protein. The exosomal IDH1 derived from 5FU-resistant cells enhanced the resistance of 5FU-sensitive cells. Metabolic assays revealed that exosomes derived from 5FU-resistant cells promoted a decrease in the level of IDH1-mediated NADPH, which is associated with the development of 5FU resistance in colorectal cancer cells. Therefore, exosomal IDH1 may be the transmitter and driver of chemoresistance in colorectal cancer and a potential chemotherapy target.

## Introduction

Colorectal cancer (CRC) is the third most deadly type of cancer in the United States 1. Current methods of treatment for CRC include surgical resection, radiotherapy, and adjuvant chemotherapy 2. 5-fluorouracil (5FU) is a conventional chemotherapy drug used for the clinical treatment of CRC that induces cell death by hindering the nucleotide metabolism of CRC cells 3. However, regardless of whether 5FU is used alone or in combination, drug resistance develops as treatment time increases 4, 5. Therefore, there is a very urgent need to solve 5FU resistance or identify a more effective treatment for CRC.

The close relationship between abnormal glucose metabolism and drug resistance is a hallmark of tumors 6. Several key enzymes involved in glucose metabolism, including PKM2, PDK1, and IDH1, are also involved in CRC development and the induction of chemotherapy resistance 7-9. Isocitrate dehydrogenase 1 (IDH1) catalyzes the production of α-ketoglutarate (α-KG) from the substrate isocitrate, which is accompanied by a reduction in NADPH 10, 11. IDH1 expression is elevated in various tumor types, including osteosarcoma, lung cancer, or gastric cancer, compared with normal tissues 12-14. The function activating mutation of IDH1 is also associated with the development of glioma and the tolerance of targeted therapy 15. As a metabolite of IDH1, α-KG is essential for maintaining dioxygenase activity in the cytoplasm 16. NADPH participates in defense against adverse oxidative reactions in cells, such as reactive oxygen species (ROS) induced by chemotherapy drugs 17. Therefore, IDH1 and its associated metabolites are beneficial for the maintainence of tumor survival and the harsh external environment. At present, the commercial IDH1 inhibitor ivosidenib (also known as AG-120) has been applied to clinical trials to treat cholangiocarcinoma and leukemia 18, 19. The role of IDH1 in the development of resistance to 5FU or other chemotherapeutic drugs in CRC has not been revealed yet.

Exosomes are nanoscale extracellular vesicles secreted by various types of cells that are widely involved in intercellular communication. They deliver their contents (including DNA, RNA, and proteins) providing instructions from the source cell to the recipient cell 20. Among tumor cells, exosomes are involved in tumorigenesis, invasion, immune response, and chemotherapy resistance and can regulate the tumor microenvironment 21. For example, exosomal miR-105 secreted by breast cancer cells can reprogram glucose metabolism in cancer-associated fibroblasts to support tumor growth 22. In CRC cells, exosomal miR-128-3p enhanced chemosensitivity to oxaliplatin 23. Our previous study found that exosomes derived from cisplatin-resistant lung cancer cells delivered PKM2 to reprogram glucose metabolism in sensitive cells and transmit drug resistance to sensitive cells 24. In CRC cells, the involvement of exosomes in cell metabolism and drug resistance development is not yet fully understood.

In this study, we found that IDH1 was highly expressed in CRC tissues and was the driver of 5FU resistance in CRC cells. Proteomics and cytology analyses were conducted to verify that 5FU-resistant CRC cells secreted exosomes that encapsulated high levels of IDH1. These exosomes derived from drug-resistant cells increased the resistance of sensitive cells to 5FU by reprogramming the reducing internal environment of sensitive cells. Our results elucidated the role of exosomal IDH1 in 5FU resistance in CRC.

## Materials and Methods

### Cell lines, plasmids, and agents

The human colorectal cancer cell line, HCT8, was purchased from the National Collection of Authenticated Cell Cultures (Shanghai, China) and cultured in RPMI-1640 (Gibco, MA, USA) containing 10% FBS (Gibco). The 5FU resistant cell line, HCT8FU, was purchased from OBIO Technology (Shanghai, China) and maintained in a medium containing 0.5 μg/ml 5FU. The IDH1 gene was cloned into a pLenti-CMV-EGFP-3FLAG-Puro vector and stably expressed in HCT8 cells using a lentivirus. The lentivirus of shRNA-NC (sense 5'-GATCCGTTCTCCGAACGTGTCACGTTTCAAGAGAACGTGACACGTTCGGAGAACTTTTTTG-3', antisense 5'-AATTCAAAAAAGTTCTCCGAACGTGTCACGTTCTCTTGAAACGTGACACGTTCGGAGAACG-3'), shRNA-IDH1-994 (sense 5'-GATCCGCAGTACAAGTCCCAGTTTGATTCAAGAGATCAAACTGGGACTTGTACTGCTTTTTTG-3, antisense 5'-AATTCAAAAAAGCAGTACAAGTCCCAGTTTGATCTCTTGAATCAAACTGGGACTTGTACTGCG-3') and shRNA-IDH1-1404 (sense 5'-GATCCGCTTCATGACCAAGGACTTGGTTCAAGAGACCAAGTCCTTGGTCATGAAGCTTTTTTG-3', antisense 5'-AATTCAAAAAAGCTTCATGACCAAGGACTTGGTCTCTTGAACCAAGTCCTTGGTCATGAAGCG-3') were used for stable IDH1 knockdown. 5FU and Ivosidenib were purchased from Selleck Chemicals (Shanghai, China). The antibodies, anti-Flag (Sigma, MO, USA), IDH1 (R&D Systems, MN, USA), CD63 (Proteintech, Wuhan, China), TSG101 (ABclonal, Wuhan, China), and β-actin (Cell Signaling Technology, MA, US) were also used in this study.

### Immunohistochemistry

Tissue microarrays (No. COC1261 and COC1601) from 125 patients with colorectal cancer were purchased from Shanghai Superbiotek Inc. All experimental procedures were approved by the Human Ethics Committee of Shanghai University of Medicine and Health Sciences. Before incubation with the anti-IDH1 (1:500) antibody, the tissue chip was treated with 3% H_2_O_2_ to block endogenous peroxidase activity. The tissue chip was incubated with the HRP-conjugated secondary antibody, and positive signals were observed using diaminobenzidine (DAB) staining. Two independent researchers scored the expression of IDH1 in each tissue based on distribution and intensity. The scores for the percentage of stained cells were 0 (0-5%), 1 (5-25%), 2 (25-50%), and 3 (50-100%). The scores for staining intensity were 0 (no staining), 1 (weak), 2 (medium), and 3 (strong). The product of the two scores were used as the expression score of IDH1 expression. Each cancer tissue was classified into either the high expression (score ≥ 6) or low expression (score <6) group.

### Cell proliferation and drug toxicity assays

HCT8 or HCT8FU cells were seeded into 96-well plates at a density of 1 × 10^4^ cells/well and treated with the required concentration of 5FU or ivosidenib. After being cultured for 24-96 h, 10 μl of CCK-8 solution (Bimake, Shanghai, China) was added. Absorbance at 450 nm was determined and used to evaluate the viability of the cells.

### Cell cycle analysis

The digested HCT8 cell suspension was fixed using cold 70% ethanol for 24 h. Then, the cells were incubated with RNase A-containing propidium iodide solution (BD Bioscience, CA, USA) at 4 °C in the dark for 15 minutes. A flow cytometer (BD) and Modfit LT software (version 3.1, Topsham, ME, USA) were used to analyze the cell cycle.

### Cell invasion assay

200 μl of DMEM resuspension containing 6×10^4^ cells was added to the upper chamber of the Transwell chamber (Corning, NY, USA). 600 μl of complete medium with serum was added to the lower chamber. After 24 h, the cells in the lower chamber that had not migrated were removed, and the migrated cells were stained with 0.1% crystal violet, and images were captured under a microscope.

### Western blotting assays

RIPA buffer (Keygenbio, Nanjing, China) was used to lyse the cells. The BCA protein assay kit (Yeason, Shanghai, China) was used to quantify total protein in the cell lysates or exosomes. Then, 40 μg of protein was separated using SDS-PAGE and transferred onto a PVDF membrane. The membrane was blocked using 5% milk for 1 hour and stained with a primary antibody overnight. Then, the membrane was incubated with an HRP-labeled secondary antibody for 1 h. An automated bioluminescence imaging system (Tanon, Shanghai, China) was used to obtain results.

### Exosomes isolation and identification

The cell culture solution was centrifuged at 120,000 g for 90 minutes using an OptimaTM XPN-100 ultracentrifuge (Beckman Coulter) to obtain exosomes derived from the HCT8 and HCT8FU cells. The exosomes were resuspended in PBS and placed on a copper grid, fixed with 1% glutaraldehyde, and treated with uranyl acetate. The images of the exosomal samples were observed under a transmission electron microscope (FEI Tecnai G2 Spirit TEM, USA) at 80 kV. ZetaView PMX 110 (Particle Metrix, Meerbusch, Germany) was used to measure the size distribution and concentration of the exosomes.

### LC/MS-MS

The exosomal proteins derived from HCT8 and HCT8FU cells were precipitated with cold acetone and dried. 100 μg of protein was digested with trypsin, and the purified peptides were separated using a Durashell C18 analytical column and identified through mass spectrometry using anh AB SCIEX nanoLC-MS/MS (Triple TOF 5600 plus). MaxQuant v1.5 database was used for proteome identification and label-free quantification. The iBAQ algorithm was used for absolute protein quantification of the exosomal samples 25.

### IDH1 activity and metabolite assay

HCT8 cells were lysed with RIPA buffer. The activity of isocitrate dehydrogenase in the cell lysates was measured using an IDH assay kit (MAK072, Sigma-Aldrich, SL, USA) as instructed by the manufacturer. A-ketoglutarate assay kit (MAK054, Sigma-Aldrich) and NADP/NADPH quantification kit (MAK038, Sigma-Aldrich) were used to measure the content of α-KG, total NADP+ and NADPH in the cell lysate. Glucose uptake was measured following previously used experimental protocols 26. The total number of cells was used as a reference for data standardization.

### Statistical analysis

All statistical analyses were performed using either Graphpad Prism 7.0 (CA, USA) and SPSS 20.0 software (IL, USA). One-way analysis of variance (ANOVA) and Bonferroni post-hoc test were used to analyze statistical differences between multiple groups. A P-value of < 0.05 was considered to indicate statistical significant.

## Results

### High expression of IDH1 can predict the malignant progression of colorectal cancer

**Figure 1:**
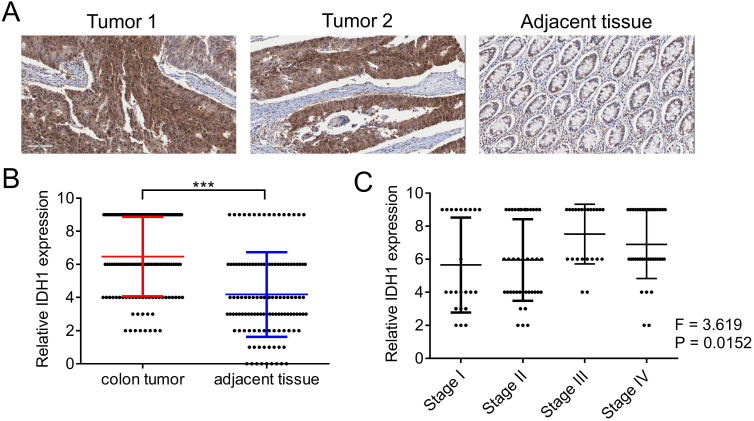
** IDH1 was highly expressed in human CRC tissues.** (A) Representative images of two tumor sections and one adjacent normal tissue section from the same patient after IHC staining with the IDH1 antibody. (B) Statistical analysis of IHC scores of IDH1 in 125 CRC tumors and adjacent normal tissues. ***, p <0.001. (C) Correlation analysis between IHC scores of IDH1 and the tumor stage of the 125 CRC tumor patients.

We first evaluated the expression pattern of IDH1 in human clinical samples. The expression of IDH1 in the tumor tissues and corresponding adjacent tissues of the 125 CRC patients was determined using immunohistochemistry (Figure 1A). Quantitative statistical analysis of the expression of IDH1 showed that IDH1 was highly expressed in CRC tissues (Figure 1B). Further analysis showed that a high-stage CRC tumor was significantly associated with a high IDH1 expression score (p = 0.0152, Figure 1C). The clinical data of the 87 CRC patients with high IDH1 expression tumors and 38 patients with low IDH1 expression tumors showed that high IDH1 expression was associated with a high-stage tumor (p < 0.001), distant metastasis (p = 0.006) and lymph node metastasis (p = 0.002, Table 1). Therefore, the high expression of IDH1 in CRC may be an indicator of malignant features.

### IDH1 promotes the proliferation of colorectal cancer cells and resistance to 5FU

**Figure 2:**
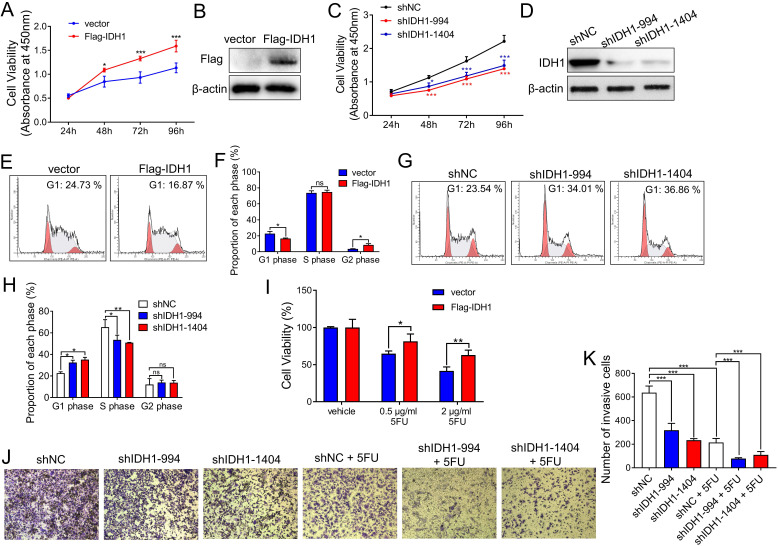
** IDH1 promotes CRC cell proliferation and resistance to 5FU.** (A) The pLenti-Flag-IDH1 plasmid and the vector plasmid were stably infected into HCT8 cells with lentivirus. The same number of HCT8 cells were plated on a 96-well plate and subjected to CCK-8 analysis to measure the number and viability of the cells. (B) Western blotting assays were used to determine Flag-IDH1 overexpression. (C) pLenti-shIDH1-994, pLenti-shIDH1-1404, and the negative control shNC plasmid were stably infected into HCT8 cells with lentivirus. Cell viability was measured daily using CCK8 analysis. (D) Western blotting assays were used to identify the knockdown effect of shIDH1-994 and shIDH1-1404. (E and F) Flow cytometry was used to analyze the cell cycle phase ratio of HCT8 cells expressing the Flag-IDH1 and the vector plasmid. (G and H) Flow cytometry was used to analyze the cell cycle phases of HCT8 cells expressing shIDH1-994, shIDH1-1404, and shNC. (I) HCT8 cells that stably overexpressed Flag-IDH1 and vector plasmids were treated with 0.5 µg/ml and 2 µg/ml 5FU for 48 h. The number and viability of the cells were measured using CCK8 analysis. (J and K) HCT8 cells stably expressing shNC, shIDH1-994 and shIDH1-1404 were used in the Transwell assays. HCT8 cells expressing shNC, shIDH1-994 and shIDH1-1404 were treated with 2 µg/ml 5FU for 48 h, and then the Transwell assays were performed. The invading cells were analyzed and counted using Image J software. NS, no significant; *, p < 0.05; **, p < 0.01; ***, p < 0.001.

Next, we explored the effect of IDH1 on tumor growth in CRC cell lines. The CRC cell line, HCT8, stably expressed Flag-IDH1. Cell proliferation assays showed that IDH1 overexpression enhanced the proliferation of HCT8 cells *in vitro* (Figure 2A and B). Meanwhile, we established two HCT8 cell lines (shIDH1-994 and shIDH1-1404) with stable IDH1 knockdown using shRNA lentiviral plasmids. The results showed that the proliferation of HCT8 cells with IDH1 knockdown using shRNA was significantly slower than that of the control group (Figure 2C and D). Cell cycle assays using flow cytometry showed that the overexpression of Flag-IDH1 decreased the proportion of HCT8 cells at the G1 phase and increased the proportion at the G2 phase (Figure 2E and F). Compared with control cells, the cell lines expressing IDH1-shRNA showed a significant increase in the proportion of cells at the G1 phase and a decrease in the proportion of cells at the S phase (Figure 2G and H), indicating that IDH1 knockdown inhibited G1-S transition in the CRC cell cycle.

The role of IDH1 in the development of CRC chemoresistance was explored. Compared with the control vector, Flag-IDH1 increased the survival rate of HCT8 cells under different concentrations (0.5, 2 µg/ml) of 5FU treatment (Figure 2I), indicating that IDH1 overexpression promoted the development of 5FU resistance. The results of the cell invasion assays showed that the invasive ability of HCT8 cells decreased after IDH1 knockdown, and that IDH1-shRNA combined with 5FU treatment further inhibited cell invasion (Figure 2J and K). These results confirm that IDH1 promotes the proliferation of CRC cells and resistance to 5FU. The combination of IDH1-knockdown and 5FU therapy can enhance the inhibition effect on the malignant behavior of CRC cells.

### Exosomes secreted by 5FU-resistant colorectal cancer cells induce drug resistance

**Figure 3:**
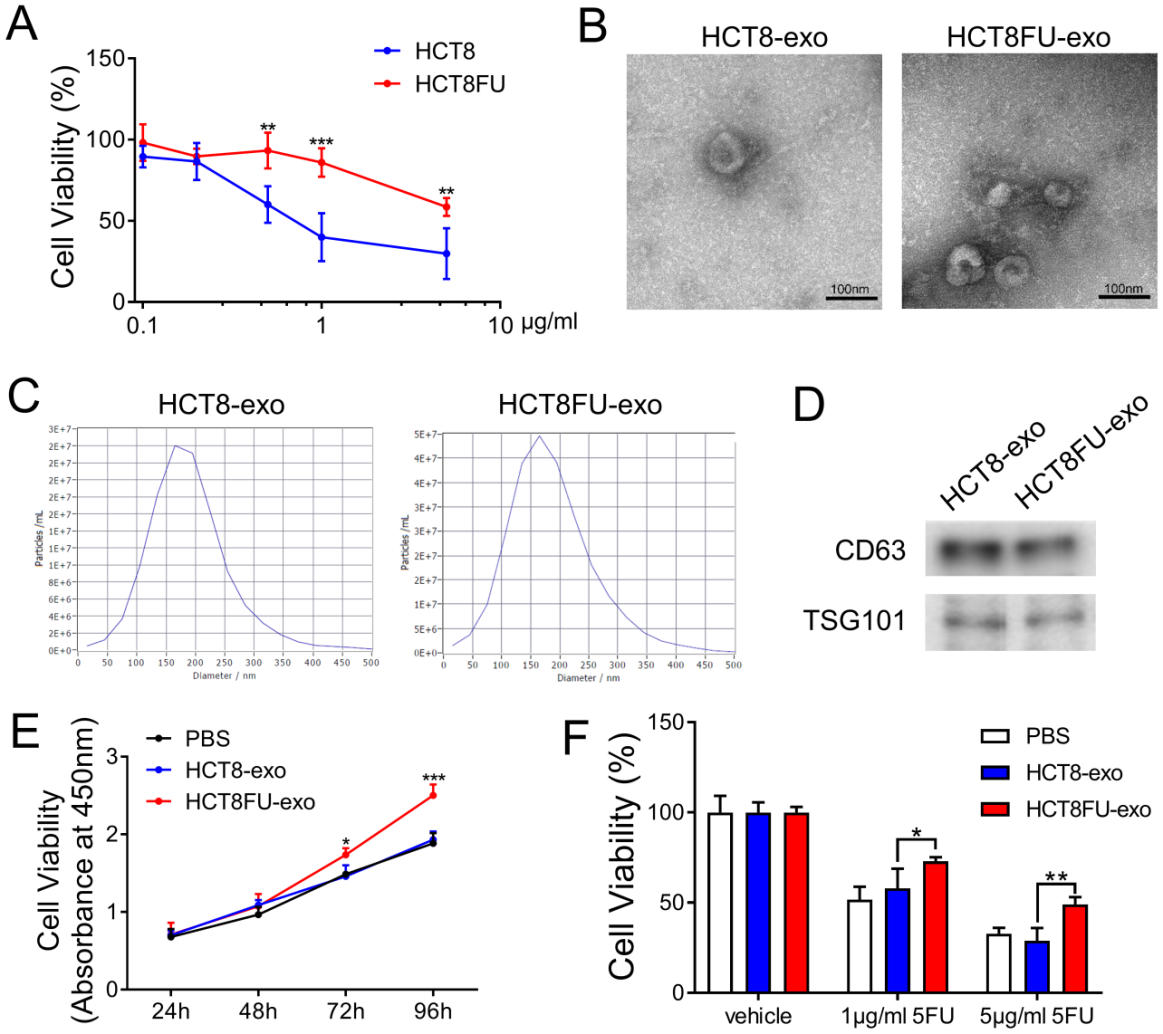
** Isolation and identification of exosomes derived from HCT8FU and promotion of 5FU resistance in sensitive cells.** (A) Cell viability analysis of HCT8 and HCT8FU cells treated with different concentrations of 5FU (0, 0.1 µg/ml, 0.2 µg/ml, 0.5 µg/ml, 1 µg/ml, and 5 µg/ml) for 48 h. (B) The morphology of HCT8-exo and HCT8FU-exo were determiend using a transmission electron microscope. Error bar, 100 µm. (C) The particle size of HCT8-exo and HCT8FU-exo were identified using NTA. (D) The expression of CD63 and TSG101 in HCT8-exo and HCT8FU-exo cells were determined using western blotting assays. (E) PBS (40 µg/ml) was co-cultured with HCT8-exo or HCT8FU-exo cells for 24, 48, 72, and 96 h, and cell growth was detected using CCK8 assays. (F) HCT8 cells were treated with 40 µg/ml HCT8-exo or HCT8FU-exo for 48 h and followed by 1 µg/ml or 5 µg/ml 5FU for 48 h. Cell viability was determined using CCK8 assays. *, p < 0.05; **, p < 0.01; ***, p < 0.001.

To study the mechanism by which 5FU resistance develops and is transmitted in colorectal cancer, we established a 5FU-resistant HCT8 cell line, HCT8FU. Cell viability assays were used to evaluate the resistance of HCT8FU cells to 5FU and to show that these cells were more resistant to 5FU than the drug-sensitive HCT8 cell line (Figure 3A). We attempted to determine whether exosomes are involved in the communication between different subgroups of colorectal cancer cells and the transmission of 5FU resistance. Exosomes (HCT8-exo and HCT8FU-exo) were isolated from HCT8 and HCT8FU cells through ultracentrifugation, and their morphology was observed using a transmission electron microscope (Figure 3B). Western blotting assays revealed that the exosomal markers, CD63 and TSG101, were expressed by HCT8-exo and HCT8FU-exo (Figure 3D), indicating successful isolation of the exosomes. Nanoparticle size analysis studies showed that these exosomes were of an average diameter of 100-250 nm (Figure 3C). Next, 40 μg/ml HCT8-exo or HCT8FU-exo was co-cultured with sensitive HCT8 cells for 24 to 96 h. The results showed that HCT8FU-exo significantly increased HCT8 cell proliferation (Figure 3E). The 5FU-sensitive HCT8 cells were incubated with 40 μg/ml HCT8-exo or HCT8FU-exo for 48 h and then exposed to 5FU for 48 h. The 5FU resistance of HCT8 cells was enhanced by HCT8FU-exo, compared with HCT8-exo (Figure 3F). These results suggest that exosomes secreted by 5FU-resistant colorectal cancer cells promoted drug resistance in sensitive colorectal cancer cells.

### High expression of IDH1 in exosomes secreted by 5FU-resistant colorectal cancer cells

**Figure 4:**
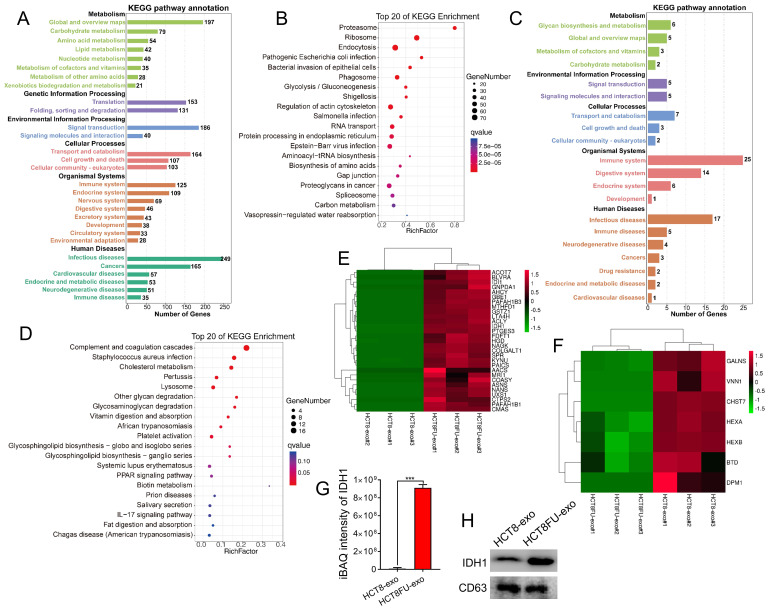
** LC-MS/MS showed that IDH1 was highly expressed in HCT8FU-derived exosomes.** (A and B) The number of KEGG pathway annotations with a high expression of proteins and the main KEGG enrichment pathways in HCT8FU-exo. (C and D) The number of KEGG pathway annotations of highly expressed proteins and the main KEGG enrichment pathways in HCT8-exo. (E and F) The heat map reflected the significantly high expression of proteins involved in glucose metabolism in HCT8FU-exo (E) and the highly expressed proteins involved in glucose metabolism in HCT8-exo (F). (G) LC-MS/MS was used to determine the iBAQ intensity of the IDH1 protein. (H) Western blotting assay data on the IDH1 protein in 20 µg of HCT8-exo and HCT8FU-exo.

LC-MS/MS was used to obtain the protein expression profile of the exosomes derived from sensitive HCT8 cells (HCT8-exo) and exosomes derived from resistant HCT8FU cells (HCT8FU-exo) to determine differences in exosomal proteins derived from 5FU-resistant colorectal cancer cells and sensitive cells. The iBAQ algorithm was used for the absolute quantification of proteins, and 1,629 differentially expressed proteins were found between HCT8-exo and HCT8FU-exo. Of these, 95 proteins were highly expressed by HCT8-exo, while 1,534 proteins were highly expressed by HCT8FU-exo. Further, KEGG enrichment analysis of the highly expressed proteins in HCT8FU-exo showed that these proteins enriched in 5FU-resistant exosomes were involved in regulation of metabolic pathways, including glycolysis and environmental information pathways (Figure 4A and 4B). The KEGG enrichment analysis showed that in HCT8-exo, immune system regulatory proteins and metabolic pathway-related proteins were highly enriched in exosomes derived from sensitive cells (Figure 4C and 4D). The proteins highly expressed by HCT8FU-exo included proteins that regulate glucose metabolism, including IDH1 (Figure 4E), while proteins involved in cell metabolism were highly expressed by HCT8-exo (Figure 4F). The absolute protein quantification of iBAQ using LC-MS/MS results showed that IDH1 was highly expressed in the exosomes of 5FU-resistant colorectal cancer cells (Figure 4G). Western blotting assays also demonstrated a high level of IDHI expression in HCT8FU-exo (Figure 4H). These results indicate that exosomal IDH1 may be involved in the development and transmission of 5FU resistance in colorectal cancer cells.

### Exosomal IDH1 promotes 5FU resistance in colorectal cancer cells

**Figure 5:**
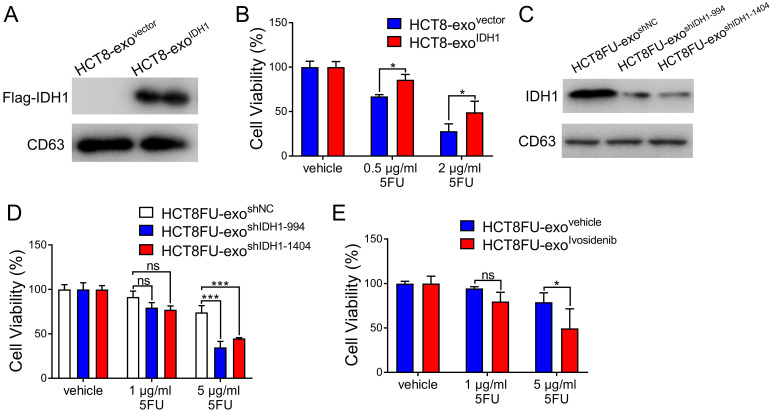
**Exosomal IDH1 promotes the resistance of colorectal cancer cells to 5FU.** (A) Exosomes (HCT8-exo^IDH1^ or HCT8-exo^vector^) from HCT8 cells that stably overexpressed Flag-IDH1 or the vector were isolated, and the expression of Flag-IDH1 in exosomes was determined. (B) HCT8 cells were treated with 40 µg/ml HCT8-exo^IDH1^ or HCT8-exo^vector^ for 48 h and then treated with 0.5 µg/ml or 2 µg/ml 5FU for 48 h. CCK8 assays were used to determine cell viability. (C) Exosomes (HCT8FU-exo^shIDH1-994^, HCT8FU-exo^shIDH1-1404,^ or HCT8FU-exo^shNC^) from HCT8FU cells that stably overexpressed shIDH1-994, shIDH1-1404 or shNC were isolated, and the expression of IDH1 in exosomes was determined. (D) HCT8 cells treated with 40 µg/ml HCT8FU-exo^shIDH1-994^, HCT8FU-exo^shIDH1-1404,^ or HCT8FU-exo^shNC^ for 48 h, followed by treatment with 1 µg/ml or 5 µg/ml 5FU for 48 h. CCK8 assays were used to determine cell viability. (E) Exosomes (HCT8FU-exo^ivosidenib^ or HCT8FU-exo^vehicle^) isolated from HCT8FU cells treated with 1 µg/ml ivosidenib or an equal volume of the vehicle for 48 h. HCT8 cells treated with 40 µg/ml exosomes for 48 h, and then treated with 1 µg/ml or 5 µg/ml 5FU for 48 h. CCK8 assays were used to determine cell viability. NS, no significant; *, p < 0.05; **, p < 0.01; ***, p < 0.001.

The viability of the HCT8 cells under 5FU exposure was used to determine whether the exosomal IDH1 derived from resistant cells could induce 5FU resistance in sensitive cells. Exosomes derived from HCT8 cells that expressed Flag-IDH1 were isolated, and exosomal IDH1 was overexpressed (Figure 5A). Exosomes with high IDH1 expression (HCT8-exo^IDH1^) and control exosomes (HCT8-exo^vector^) were co-cultured with HCT8 cells and then exposed to 5FU. The results showed that exosomes with elevated IDH1 expression significantly promoted 5FU resistance in HCT8 cells (Figure 5B). Exosomes derived from HCT8FU cells (HCT8FU-exo^shIDH1-994^ and HCT8FU-exo^shIDH1-1404^) with stable IDH1 knockdown were also extracted (Figure 5C) and co-cultured with HCT8 cells. Exosomes with low levels of IDH1 expression enhanced the sensitivity of HCT8 cells to 5FU (Figure 5D). The commercial IDH1 inhibitor, ivosidenib, was used to treat HCT8FU cells, and then exosomes were extracted (HCT8FU-exo^ivosidenib^) and used to increase the drug sensitivity of HCT8 cells (Figure 5E). Together, these results indicate that drug-resistant colorectal cancer cells secrete exosomes that confer 5FU resistance to sensitive cells by delivering IDH1.

### Exosomal IDH1-mediated glycometabolism reprogramming is involved in 5FU resistance

**Figure 6:**
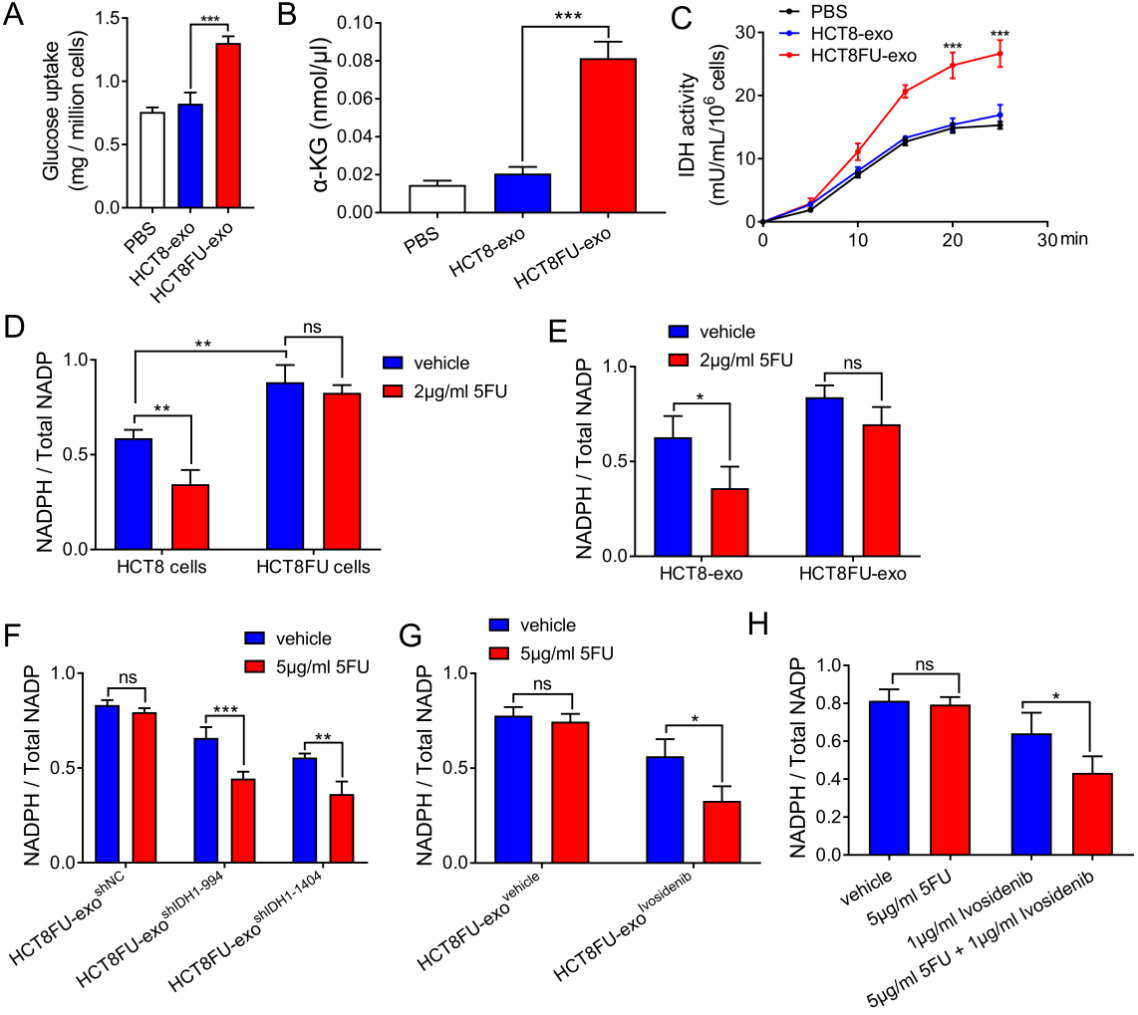
** Exosomes derived from drug-resistant cells promote resistance in 5FU sensitive cells through IDH1-dependent metabolic pathways.** (A) HCT8 cells were treated with 40 µg/ml HCT8-exo or HCT8FU-exo for 48 h, and then glucose uptake assays were performed. (B) The level of a-KG in the cell lysate of HCT8 cells co-cultured with exosomes. (C) HCT8 cells co-cultured with lysed exosomes. IDH1 activity curves were used for 25 minutes of measurements. (D) HCT8 and HCT8FU cells treated with 2 µg/ml 5FU or an equal volume of the vehicle for 48 h, and levels of intracellular NADPH and total NADP+. (E) HCT8 cells treated with 40 µg/ml HCT8-exo or HCT8FU-exo for 48 h, followed by treatment with 2 µg/ml 5FU or the vehicle for 48 h. The levels of intracellular NADPH and total NADP+. (F and G) HCT8 cells treated with 40 µg/ml HCT8FU-exo^shIDH1-994^, HCT8FU-exo^shIDH1-1404^, HCT8FU-exo^shNC^ (F), HCT8FU-exo^ivosidenib^ or HCT8FU-exo^vehicle^ (G) for 48 h, and then treated with 5 µg/ml 5FU or vehicle for 48 h. Levels of intracellular NADPH and total NADP+. (H) HCT8FU cells treated with 1 µg/ml ivosidenib, 2 µg/ml 5FU, a combination of the two drugs or equal volume vehicle for 48 h. Levels of intracellular NADPH and total NADP+. NS, no significant; *, p < 0.05; **, p < 0.01; ***, p < 0.001.

The exosomes derived from drug-resistant cells that delivered IDH1 accelerated the glucose metabolism of the sensitive cells that took them up to achieve 5FU resistance. HCT8 cells that ingested HCT8FU-exo showed significantly increased glucose consumption than those that ingested HCT8-exo (Figure 6A). HCT8FU-exo also increased levels of intracellular α-KG production in HCT8 cells, compared with HCT8-exo (Figure 6B). Monitoring of isocitrate dehydrogenase activity showed that IDH1 activity was elevated in HCT8 cells that ingested HCT8FU-exo (Figure 6C). IDH1 mediates the production of the intracellular reducing metabolite, NADPH, while previous studies have also indicated that NADPH can neutralize tumor cell damage caused by chemotherapy drugs 17, 27. In 5FU-resistant HCT8FU cells, the basal level of NADPH was higher than that of sensitive HCT8 cells (Figure 6D). 5FU significantly decreased the level of NADPH in HCT8 cells, which gave evidence that the cytotoxicity of 5FU was accompanied by low levels of NADPH (Figure 6D). When HCT8FU-exo was co-cultured with HCT8 cells treated with 5FU, intracellular NADPH levels did not show a significant change (Figure 6E). To reverse the effect of exosomal IDH1 on the production of NADPH, we used 5FU to treat HCT8 cells that had internalized HCT8FU-exo^shIDH1-994^, HCT8FU-exo^shIDH1-1404^ (Figure 6F) or HCT8FU-exo^ivosidenib^ (Figure 6G). The results showed that 5FU decreased NADPH levels in HCT8 cells co-cultured with HCT8FU-exo, whose IDH1 activity or content was inhibited (Figure 6F and 6G). To revert resistance caused by exosomal IDH1, the IDH1 inhibitor, ivosidenib, and 5FU co-treatment were used. Compared with 5FU, the combination of ivosidenib and 5FU significantly decreased NADPH levels in HCT8FU cells (Figure 6H). Our data confirmed that drug-resistant cells secrete IDH1-containing exosomes that increase the intracellular NADPH levels of sensitive cells that trigger drug resistance.

## Discussion

Since its introduction in 1957, 5FU has been a first-choice drug for CRC chemotherapy. However, resistance to 5FU develops as treatment time increases, disrupting its successful anti-tumor effect. It is generally believed that 5FU resistance is associated with enhanced DNA repair and accelerated drug metabolism. The catabolism of 5FU relies on dihydropyrimidine dehydrogenase (DPD). Abnormal glucose and lipid metabolism and histone-related transcription can increase DPD expression in CRC cells, leading to 5FU resistance 28. Clinically, 5FU is often used in combination with oxaliplatin, irinotecan, and other drugs to increase its therapeutic effect 29, but the development of drug resistance cannot be avoided. Studies have shown that DNA polymerase ζ REV7 upregulation contributes to 5FU and oxaliplatin resistance when used in combination 30. Therefore, other types of solutions are needed to avoid the development of 5FU resistance in CRC.

The Warburg effect, which is based on abnormal glucose metabolism, promotes drug resistance in CRC. Chemotherapy in CRC also reprograms the metabolic pathways of surviving tumor cells. These surviving cells, namely drug-resistant cells, undergo a series of changes that enhance their malignant phenotypes, including a tumor stem cell-like phenotype, which leads to tumor metastasis and tumor immunosuppression 31, 32. PDK2 was highly expressed in 5FU resistant cells, while dichloroacetate or miRNA targeting PDK2 enhanced CRC cell sensitivity to 5FU 33. LncRNA XIST promoted glycolysis and 5FU chemoresistance in CRC by increasing PKM2 levels 34. These metabolic enzymes act as direct regulators of glucose metabolism and are responsible for CRC chemoresistance. Our study revealed that IDH1, a key enzyme involved in glucose metabolism, enhanced CRC cell growth and 5FU resistance. Therefore, IDH1-related tumor metabolism may be a target for the treatment of 5FU-resistant CRC.

Previous studies have confirmed that IDH1 mediates chemoresistance in ovarian cancer and endometrial cancer 35, 36, while there is a lack of research on chemoresistance in colorectal cancer. Most studies have focused on the activation of IDH1 R132 mutations, which are often found in many types of tumors. IDH1 R132 promotes tumor progression and treatment tolerance, which can be mainly attributed to its abnormal metabolite 2-hydroxyglutarate. Anti-apoptotic genes can be targeted to inhibit tumors carrying IDH1 mutations 37. A previous study 38 and our data showed that unmutated IDH1 was highly expressed in tumor tissues and was accompanied by an increase in the production of α-KG and NADPH. This indicates that the upregulation of IDH1 can reprogram the metabolic state of tumor cells, enhancing tumor growth and chemoresistance. Despite being a subtype of isocitrate dehydrogenase, IDH1 is not involved in the oxidative phosphorylation, which takes place in mitochondria. IDH1 is mainly located in the cytoplasm and peroxisomes and maintains the reducing internal environment by producing reducing substances, such as NADPH 39. We speculated that IDH1 was delivered to sensitive CRC cells via exosomes, which in turn increased the viability of cells under 5FU treatment through the accumulation of NADPH. This novel form of tumor metabolic reprogramming will have an effect on tumor phenotype.

We found that exosomes transmitted the IDH1 protein to sensitive cells and promoted CRC cell resistance to 5FU. A previous study revealed that the 5FU-resistant CRC cell line RKO/R secretes exosomes that enhance 5FU resistance in RKO cells. This process relies on the phosphorylated STAT3 protein in exosomes 40. Therefore, exosomal proteins are critical disseminators of 5FU resistance in CRC cells. Apart from the exosomes secreted by drug-resistant cells, other components in the tumor microenvironment also produce other 5FU resistance-inducing exosomal proteins that are delivered to CRC cells. In the tumor microenvironment, cancer-associated fibroblasts (CAFs) secrete exosomes that carry Wnt, which promote CRC cell differentiation into tumor stem cells 41. These exosomal proteins are regarded as effective targets to reverse CRC resistance, and further research is needed to explore the mechanisms involved. Apart from the exosomal proteins derived from resistant cells that were identified through our exosomal proteomics results, other exosomal proteins that cause 5FU resistance in CRC cells also need to be identified. Additionally, CAFs also transmit miRNA-encapsulated exosomes to CRC cells to enhance tumor cell invasiveness and 5FU resistance 42. Multiple exosomal miRNAs, including miRNA-125b, miR-21, and miR-210, are involved in CRC cell resistance to 5FU and serve as biomarkers of the malignant phenotype and the therapeutic effect of CRC 43-45. Therefore, the complete elucidation of exosomal proteins or RNAs that cause 5FU resistance will allow for the advancement of tumor treatment methods.

In summary, our data proves that enhanced expression of IDH1 in CRC tissues can mediate the rapid growth of CRC cells and the occurrence of chemoresistance. Exosomes secreted by 5FU-resistant CRC cells contribute to the growth of sensitive CRC cells and the development of 5FU resistance. This resistance depends on the communication between resistant and sensitive cells, which is mediated by exosomal IDH1. Exosomal IDH1 can increase the concentration of metabolites of α-KG and NADPH in sensitive cells, ensuring CRC cell survival in a 5FU environment. IDH1 and exosomal IDH1 are potential targets for CRC treatment and drug resistance.

## Figures and Tables

**Table 1:** Correlation between IDH1 expression and clinical data of CRC patients

	All Cases	High LDH1 expression	Low LDH1 expression	*p*-value
Participants	125	87	38	
**Sex**				0.937
Male	75	52	23	
Female	50	35	15	
**Age**				0.859
<60 years	64	45	19	
≥60 years	61	42	19	
**Tumor Stage**				<0.001*
I	23	10	13	
II	41	23	18	
III	23	21	2	
IV	38	33	5	
**Distant metastasis**				0.006*
Y	38	33	5	
N	87	54	33	
**Lymph Node Metastasis**				0.002*
Y	49	42	7	
N	76	45	31	

*p < 0.05.

## Abbreviations

CRC: colorectal cancer
IDH1: isocitrate dehydrogenase 1
α-KG: α-ketoglutarate
NADPH: reduced form of nicotinamide-adenine dinucleotide phosphate
